# Humidity Sensing Ceria Thin-Films

**DOI:** 10.3390/nano12030521

**Published:** 2022-02-02

**Authors:** Vilko Mandić, Arijeta Bafti, Luka Pavić, Ivana Panžić, Stanislav Kurajica, Jakov-Stjepan Pavelić, Zhen Shi, Katarina Mužina, Ivana Katarina Ivković

**Affiliations:** 1 Faculty of Chemical Engineering and Technology, Marulićev trg 20, 10000 Zagreb, Croatia; ipanzic@fkit.hr (I.P.); stankok@fkit.hr (S.K.); jpavelic@fkit.hr (J.-S.P.); kmuzina@fkit.hr (K.M.); imunda@fkit.hr (I.K.I.); 2 Ruđer Bošković Institute, Bijenička Cesta 54, 10000 Zagreb, Croatia; luka.pavic@irb.hr; 3 College of Materials and Environmental Engineering, Hangzhou Dianzi University, 1158, No.2 Street, Hangzhou 310018, China; zhenshi@hdu.edu.cn

**Keywords:** thin-films, relative humidity sensors, ceria nanoparticles, solid-state impedance spectroscopy, grazing incidence X-ray diffraction (GIXRD), atomic force microscopy (AFM), field emission scanning electron microscopy (FESEM)

## Abstract

Lowering the constitutive domains of semiconducting oxides to the nano-range has recently opened up the possibility of added benefit in the research area of sensing materials, in terms both of greater specific surface area and pore volume. Among such nanomaterials, ceria has attracted much attention; therefore, we chemically derived homogeneous ceria nanoparticle slurries. One set of samples was tape-casted onto a conducting glass substrate to form thin-films of various thicknesses, thereby avoiding demanding reaction conditions typical of physical depositions, while the other was pressed into pellets. Structural and microstructural features, along with electrical properties and derivative humidity-sensing performance of ceria thin-films and powders pressed into pellets, were studied in detail. Particular attention was given to solid-state impedance spectroscopy (SS-IS), under controlled relative humidity (RH) from 30%–85%, in a wide temperature and frequency range. Moreover, for the thin-film setup, measurements were performed in surface-mode and cross-section-mode. From the results, we extrapolated the influence of composition on relative humidity, the role of configuration and thin-film thickness on electrical properties, and derivative humidity-sensing performance. The structural analysis and depth profiling both point to monophasic crystalline ceria. Microstructure analysis reveals slightly agglomerated spherical particles and thin-films with low surface roughness. Under controlled humidity, the shape of the conductivity spectrum stays the same along with an increase in RH, and a notable shift to higher conductivity values. The relaxation is slow, as the thickness of the pellet slows the return of conductivity values. The increase in humidity has a positive effect on the overall DC conductivity, similar to the temperature effect for semiconducting behavior. As for the surface measurement setup, the thin-film thickness impacts the shape of the spectra and electrical processes. The surface measurement setup turns out to be more sensitive to relative humidity changes, emphasized with higher RH, along with an increase in thin-film thickness. The moisture directly affects the conductivity spectra in the dispersion part, i.e., on the localized short-range charge carriers. Moisture sensitivity is a reversible process for thin-film samples, in contrast to pellet form samples.

## 1. Introduction

In various fields such as industry, agriculture, environmental protection, health monitoring, and household use, the application of humidity sensors is necessary [1]. The inherent characteristics of humidity sensors should include high sensitivity and selectivity, as well as a wide detection range, along with easy and affordable production. [2]. Various materials can be used for the constitutive parts of humidity sensors, but the advantages of metal oxides lie in their stability (mechanical, chemical, and physical), thermally broad operational range, and fast response and recovery [2]. For example, CeO_2_ is a metal oxide used as a humidity sensor, but also as a catalyst, a solid electrolyte in fuel cells, a high-temperature ceramic, coating, etc. [3,4,5,6]. For sensing application, the composition of materials is, of course, critical, but morphologically the nanometer dimension of a particle constituent also plays a beneficial role in the advancement of sensing properties, by providing a large specific surface area and pore volume [7]. Therefore, the reports on better sensing performance of nanostructured ceria compared to the bulk material come as no surprise [8,9]. For the preparation of ceria thin-films, a variety of physical deposition methods has been reported [9,10]. Several reports of relatively demanding methods, such as pulsed laser deposition or magnetron sputtering, stand out [10]. Among demanding chemical depositions, it was shown that chemical vapor deposition can be used [11]. Provided the course of the deposition is well optimized, equally successful thin-films can be derived by the much simpler and affordable tape casting deposition of previously precipitated nanoparticles [12].

The reason behind the favorable properties of ceria for sensing lies in their electronic structure: Ce^3+^ ions and oxygen vacancies. Specifically, ceria displays a high charge density, due to the high positive charge and small ionic radius of Ce^4+^ ions. For nanostructured ceria particles, such a strong electric field can also increase the ionization of water molecules on the surface and affect the deeper physiosorbed water [8]. As a consequence, the nano-ceria display great oxygen storage and release capacity via simple Ce^4+^/Ce^3+^ redox cycles [13,14,15]. The resistance in ceria films decreases exponentially along with increasing humidity; consequently, an ion-conductivity mechanism dominates the sensing [7,16,17]. As previously stated, nano-structuring plays a considerable role in the performance of a sensor; from the point of view of ceria sensors, Fu et al. investigated hydrothermally prepared ceria nanowires due to their fast sensing response [8] and in terms of their implementation in fast humidity sensors. It was found that the variation in the number of water molecules adsorbed on the surface when changing relative humidity also controlled electrical behavior [18]. On the other hand, the transport of the proton ions on the sample surface is unrestricted due to Grotthuss’s chain reaction [19,20]. Reports on doped ceria nanoparticles, regarding humidified atmosphere monitoring adsorption behavior and electrical conductivity, are also available [20]. Under a humidified atmosphere, proton conductivity of dense monocrystalline ceria was in the range 10^−7^ and 10^−8^ S cm^−1^ at ~50 °C. The proton conductivity decreased along with the increase in temperature. Excellent recovery and repeatability for the ceria sensor with the linear response are reported for a wide span of relative humidities, from 11–97%, for sensors prepared by the microwave-assisted method [21]. Moreover, the structural heterogeneity and large surface-to-volume ratio of sintered ceria nanoparticles lead to high values of the dielectric constant in the low-frequency range for the sintered ceria nanoparticles, due to large surface to volume ratio [22]. AC conductivity is constant up to 175 °C, after which it starts to be temperature-sensitive, followed by an increase as the temperature continues to rise. Data on the impedance spectroscopy approach is extremely scarce in the literature for ceria thin-film humidity sensors, especially bearing in mind the different characterization setups and geometries. Moreover, despite the different geometries, the available literature mainly focuses on the resistance response to an increase in humidity, while the frequency of the measurement is fixed [23]. In line with this, there is a need to study this sensing material in a wide range of frequencies and temperatures.

In this work, we present the development of ceria humidity sensor materials in thin-film and bulk configurations. Through a wide range of structural, microstructural, and electrical characterizations, with a particular focus on Solid-State Impedance Spectroscopy (SS-IS), we monitor the influence of the various synthetic and deposition parameters on the humidity sensing properties, as well as other key properties of the derived thin-films. Interestingly we have been able to dedicate measuring setups to differentiate bulk, surface and cross-section properties, in a wide frequency range from 0.1 to 10^6^ Hz. Therefore, the discussion particularly focuses on the functional aspects of different sample and measurement geometry configurations, as well as the influence of film thickness on subsequent electrical properties.

## 2. Materials and Methods

### 2.1. Preparation

**Figure 1:**
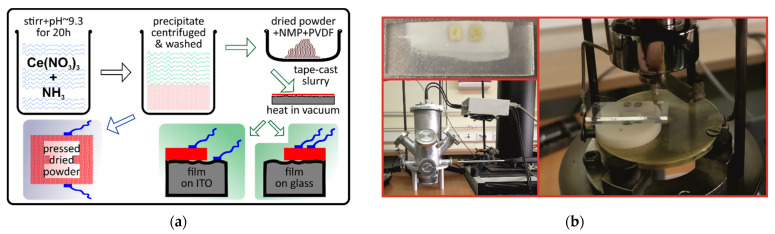
(**a**) Scheme of the synthesis, processing and characterization setups; (**b**) CeO_2_ sample in the form of thin-film on FTO glass substrate with gold electrodes (surface measurements setup, left up), sample connected to the instrument cell (left down), and setup for relative humidity measurements (right).

Ceria powder synthesis has been reported elsewhere [24]. Briefly, 200 mL of 0.2 mol solution of cerium(III) nitrate hexahydrate (p.a., Merck, Darmstadt, Germany) in deionized water were heated to 70 °C and stirred at a rate of 500 rpm. Then 100 mL of 3M ammonia solution (p.a., Alkaloid, Skopje, Northern Macedonia) were added, causing immediate precipitate formation. The stirring speed was increased to 700 rpm and the reaction was carried out for 5 min. The pH value of the reaction mixture was kept at ~9.3 by adding a few drops of concentrated ammonia solution. The formed precipitate was aged for 20 h, after which it was centrifuged, washed with water and ethanol with the help of sonication, and subsequently dried at 60 °C. The obtained product was a fine yellow powder. For the preparation of the thin-film samples, a solution of polyvinyl diethyl fluoride (PVDF, Sigma Aldrich, St. Louis, MO, USA) dissolved in N-methyl pyrrolidine (NMP, 99%, Thermo Fisher Scientific, Waltham, MA, USA) was used as a binder. The powder was mixed with the solution to form a thick suspension. This suspension was consolidated on glass slides by a doctor blade with a clearance of 10, 20, 40, and 80 μm. Two types of glass slides were used, regular microscope slides and fluorine tin oxide glass (FTO, Ossila, Sheffield, UK). Glass slides were washed in acetone and ethanol with the help of sonication before being used as a thin-film substrate. The formed ceria films were dried under a vacuum at 100 °C overnight. A scheme of the synthesis, processing and characterization setups are included in Figure 1a.

### 2.2. Characterization

Powder X-ray diffraction (PXRD) analysis was performed using an XRD 6000 diffractometer (Shimadzu, Kyoto, Japan) with CuKα radiation. The obtained data were collected in the range of 5–100°2θ in the step mode (step of 0.02°2θ), and the retention time per step was 0.6 seconds. The average crystallite size was calculated from the (200) peak using the Scherrer equation: *d* = *kλ*/(*β*cos*θ*), in which *d* represents the average size of the crystallite, *k* is the Scherrer constant which in a given case of spherical crystallites of cubic symmetry is 0.94, *λ* is the wavelength of CuKα X-ray radiation and is 0.15418 nm, *β* is the width at half the height of the diffraction peak corrected for instrumental broadening, and *θ* is the Bragg angle. The X-ray experiment using dedicated synchrotron X-ray apparatus was performed at the Materials Characterization by X-ray diffraction (MCX) beamline of the Elettra synchrotron radiation facility (Trieste, Italy) using 2.4 GeV synchrotron radiation delivered using bending magnets. The incident beam energy was 8 keV, corresponding to CuKα_1_ radiation. The apparatus rests on a four-axis Huber goniometer equipped with a fast scintillator detector in Bragg-Brentano grazing incidence setup (GIXRD) [25]. The thin-film sample was glued on a flat sample holder and adjusted in position using theta-scan (tilting) and z-scan (height). For these measurements, a CeO_2_ thin-film was used at different incidence angles.

Scanning electron microscopy (SEM) was performed using a high-resolution scanning electron microscope JSM-7000F (JEOL, Tokyo, Japan) at a voltage of 10 kV.

N_2_ adsorption-desorption isotherms were recorded on Micromeritics ASAP-2000 (Micromeritics Instruments Corp., Norcross, GA, USA) at 77 K. The sample was previously degassed at 100 °C in a dynamic vacuum of 7 mPa. Desorption data were used to calculate the specific surface area of the powder using the Brunauer-Emmett-Teller (BET) method, while the Barret-Joyner-Hallenda (BJH) method was used to calculate the pore size distribution. 

The UV-Vis spectra of the powder were collected utilizing QEPRO (Ocean Insight, Duiven, The Netherlands) spectrometer with DH-2000-DUV and ISP-50-8-R-GT integrating sphere. The diffuse reflectance spectrum was recorded in the wavelength range 300–900 nm and BaSO4 was used as a reference. Band-gap energy was calculated via Tauc equation, (*αhν*)^n^ = *B*(*hν*–Eg), where α is extinction coefficient, *h* is the Planck’s constant and *ν* is frequency; the exponent n is associated with the electronic transition in the course of optical absorption process and is theoretically equal to ½ and 2 for indirect and direct allowed transitions, respectively; *B* is a constant and *E*g is band-gap energy [26]. The diffuse reflectance spectra were transformed via Kubelka-Munk transformation of the measured spectrum to Kubelka-Munk function, F(*R*) = (1–*R*)^2^/(2*R*), where F(*R*) is proportional to the extinction coefficient (*α*) and R is the reflectance of the “infinitely thick” layer of the solid [2]. The bandgap energy, *E*g, was assessed by plotting (F(*R*)*hν*)^n^ vs. photon energy (*hν*), the so-called Tauc’s plot, followed by extrapolation of the linear region onto the energy axis.

Atomic force microscopy (AFM) characterization was conducted using CoreAFM (Nanosurf, Liestal, Switzerland) under ambient conditions. For the images acquisition, non-contact (tapping) mode was used. The Tap300Al-G probe with nominal resonant frequency of 300 kHz and tip radius of less than 10 nm turned out to be the best for this characterization. The scanning parameters were initialized using a setpoint of 35 or 55 nN of contact force with an acquisition time of 0.78 s on a 10 × 10 μm surface. Images were processed with the Gwyddion program [27].

The electrical properties of the prepared CeO_2_ samples, in the form of thin-films and pellet samples, were studied by solid-state impedance spectroscopy. An impedance analyzer Alpha-A Dielectric Spectrometer, (Novocontrol Technologies, Montabaur, Germany) was used to measure impedance at controlled relative humidity (RH) from 30% to 85%, and for selected samples measurements were also made in a wide temperature and frequency range. A closed in-situ chamber was used to measure impedance in the humidified atmosphere, where humidity conditions were fixed inside, on behalf of the use of specific salts. The humidity is expressed as relative humidity with respect to the capacity of the air to embed the water vapor. For these measurements, both bulk CeO_2_ material and CeO_2_ thin-films with thickness variation were used. One set of measurements was performed on a polycrystalline sample pressed into a pellet of the approximate thickness of 1 mm and 5 mm in diameter. For the electrical contacts, gold electrodes, 3.8 mm in diameter, were sputtered onto both sides of the pellets using a Sputter Coater SC7620 (Quorum Technologies, Laughton, UK). The impedance temperature-dependent measurements were made in the nitrogen atmosphere over a frequency range from 0.1 Hz to 0.1 MHz and in the temperature range from 30 to 210 °C with a step of 30 °C. A frequency sweep at each temperature was repeated twice. The temperature was controlled with an accuracy of ±0.2 °C. Moreover, the measurements were performed in isothermal mode at the ambient temperature in a wide range of relative humidity (RH) from 30% to 85%, which was obtained using various salts (their saturated aqueous solutions). For the thin-film configuration, measurements were made in the frequency range from 0.1 Hz to 0.1 MHz at a voltage of 20 mV at ambient temperature in a wide range of RH from 30% to 85%. Two sets of thin-film measurements were performed: (i) surface and (ii) cross-section. In this case, for the electrical contacts, gold electrodes (surface 4 × 2 mm) were deposited 4 mm apart on the sample surface using a Sputter Coater SC7620 (Quorum Technologies, Laughton, UK). To make a connection with the instrument cell, for (i) surface measurements, platinum wires were attached to the surface of gold pads, whereas for (ii) cross-section measurements, platinum wires (Advent Research Materials Ltd, Oxford, UK) were attached to the surface of the FTO glass substrate and one gold pad on thin-film, thus enabling electrical characterization of the film in the cross-sectional geometry, in addition to the surface. In order to check the reliability of the results for both thin-film samples and pellets, measurements were performed three times (each time on different sample areas and samples, respectively) under identical experimental conditions (Figure 1b). The experimental data were analyzed by equivalent circuit modelling using the complex non-linear least-squares (CNLLSQ) fitting procedure and the corresponding parameters were determined using WinFit software, Version 3.2 (Novocontrol Technologies, Montabaur, Germany). The procedure is based on the fitting of the experimental impedance to a suitable equivalent circuit model. The values of the resistance obtained from the fitting procedures, *R*, and electrode dimensions (*d* is sample thickness and *A* is electrode area) were used to calculate the DC conductivity, *σ*_DC_ = *d*/(*R* × *A*).

## 3. Results

### 3.1. Structural and Microstructural Analysis of Powders

**Figure 2:**
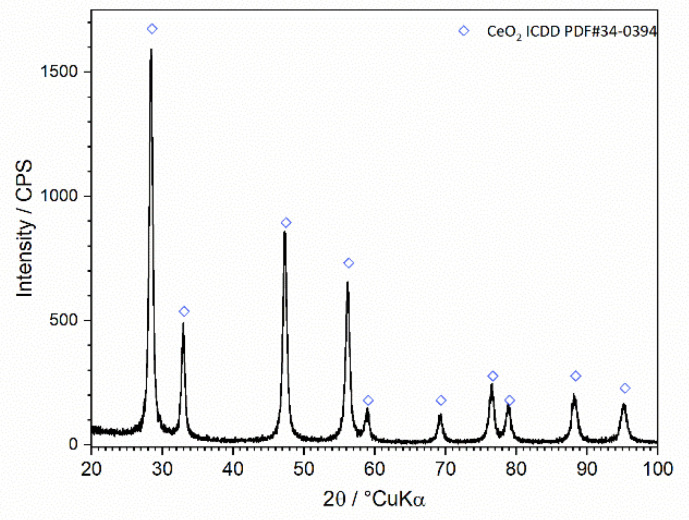
Diffraction pattern of the prepared ceria powder sample.

The diffraction pattern of the prepared CeO_2_ powder sample is shown in Figure 2. All diffraction peaks, with angles and intensities, correspond to fluorite cubic structure ceria (ICDD PDF#34-0394). High purity powders were obtained; no other peaks were observed, indicating the presence of additional phases. The ceria phase yields crystallites in the nanometer size range as indicated by the moderately broadened peaks. The average crystallite size was calculated through the Scherrer equation from the (220) peak, broadening to 23 nm.

**Figure 3:**
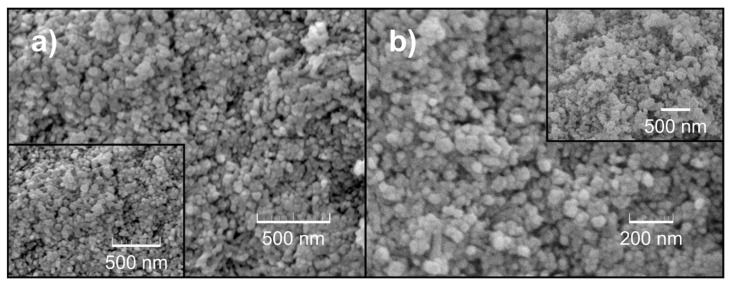
FE-SEM micrographs of the prepared powder CeO_2_ sample: (**a**) smaller magnification; (**b**) greater magnification.

In Figure 3 micrographs of the same sample at different magnifications are shown. In Figure 3a, small, relatively isotropic particles could be observed, with relatively narrow size distribution, but fairly agglomerated. On the other hand, Figure 3b gives a better perspective on the spherical particles. However, the grain boundaries remain relatively unclear. Thus the image analysis software was unable to accomplish the determination of particle size; however, it could be estimated that the average particle size is slightly greater than 23 nm.

**Figure 4:**
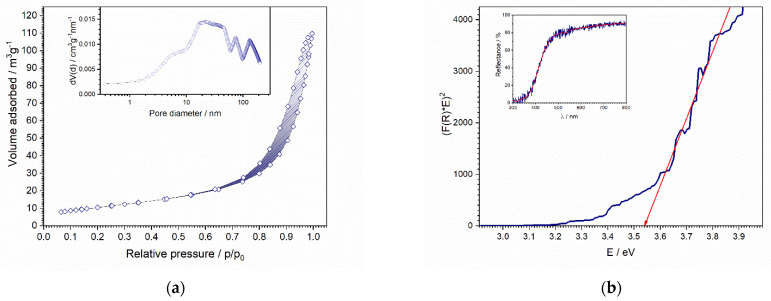
(**a**) N_2_ adsorption-desorption isotherms of the prepared powder; (**b**) Tauc’s plot for direct transition; and Inset: UV–Vis diffuse reflectance spectra (inset) of the prepared sample.

The N_2_ adsorption-desorption isotherms and pore size distribution are presented in Figure 4a. The isotherms according to the IUPAC classification belong to type IV with a H3 hysteresis loop, which refers to mesoporous materials and indicates the formation of particle aggregates [28]. The specific surface area of the powder sample was calculated to be 38.7 m^2^ g^−1^ and a pore volume of 0.15 cm^3^ g^−1^, while the average pore size was calculated as 15.7 nm. It is shown that the pore volume plays a crucial role in terms of sensor performance. While large pore volume (i.e., high surface area) is beneficial for humidity sensor sensitivity due to the ease with which water (H_3_O^+^ ions) passes easily through the pores and influences electrical conductivity, small pore size is important, to define the detection limit of the sensor. By decreasing the pore size, the quantity of water intake is limited and the influence on electrical conductivity is reduced and controlled, resulting in a lower limit of detection [1,29].

The bandgap of the powder was estimated from UV-Vis spectroscopy (Figure 4b). The inset in Figure 4b shows the UV–Vis spectrum of the powder. The spectrum shows absorbance in the UV region and reflectance in the visible region. The ceria absorption edge arises from direct transitions from the top of the valence band (O 2p states) to empty 4f-shells of Ce^4+^ in CeO_2_. [30,31]. Therefore, (F(R)hν)^2^ has been plotted versus incident photon energy (hν) (Tauc’s plot) and, by extrapolation of the linear portion onto the energy axis, the value of the direct bandgap energy was obtained as 3.54 eV (Figure 4b). The obtained value is considerably greater than the bulk CeO_2_ literature value (3.19 eV) [32], i.e., a blue shift of the absorption band is observed. Such behavior is explained by the alteration of electron energy levels from continuous in bulk to discrete in nanoscale ceria. Blueshift is then a consequence of the fact that electrons have to cross a greater distance in terms of energy, and produce photons of greater energy and shorter-wavelength [33].

### 3.2. Structural and Microstructural Analysis of Thin-Films

**Figure 5:**
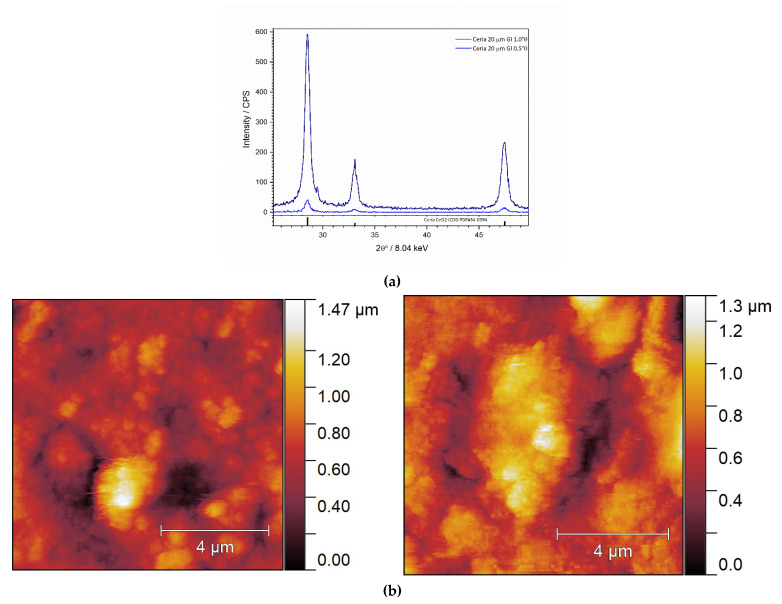
(**a**) Grazing incidence X-ray diffraction pattern of thin-film sample with 20 μm nominal thickness. (**b**) AFM micrographs of thin-film samples with 40 μm (**left**) and 80 μm (**right**) thickness, where Sq for 40 μm thick sample is 272.79 nm, and for 80 μm thick sample is 287.67 nm, respectively.

In the grazing incidence diffraction patterns (Figure 5a), the presence of a single phase can be observed which is attributed, as already observed for powder CeO_2_ sample, to cerium(IV) oxide (ICDD PDF #34-0394). The depth profiling at different angles of incidence points to the homogeneous ceria phase which is fully in concordance with the tape-casting deposition of previously prepared particulate material.

Figure 5b shows AFM micrographs of the thin-film with a thickness of 40 and 80 μm, respectively. Based on micrographs, it could be observed that thin-film is crack-free and has a granular surface. Several thin-film samples were examined to determine the range of surface roughness. The surface roughness (*S*q) is determined by squaring each value of Z (height) in the sample and rooting the arithmetic mean of these values. The roughness is thus calculated as the arithmetical average of the absolute heights in the entire height profile of the sample so that the presence of a smaller number of larger deviations can affect the roughness. For the cerium (IV) oxide samples, the topography results are consistent with the film thickness and surface roughness: sq 272.79 nm for the 40 µm thin-film and 287.76 nm for the 80 µm thin-film, which shows that the sample of 40 µm thickness consists of smaller sized particles (or, to be precise, agglomerates) which form a smoother surface than the 80 µm sample.

### 3.3. Electrical Properties

#### 3.3.1. Thick Pellet Configuration: Temperature-Dependent Conductivity

**Figure 6:**
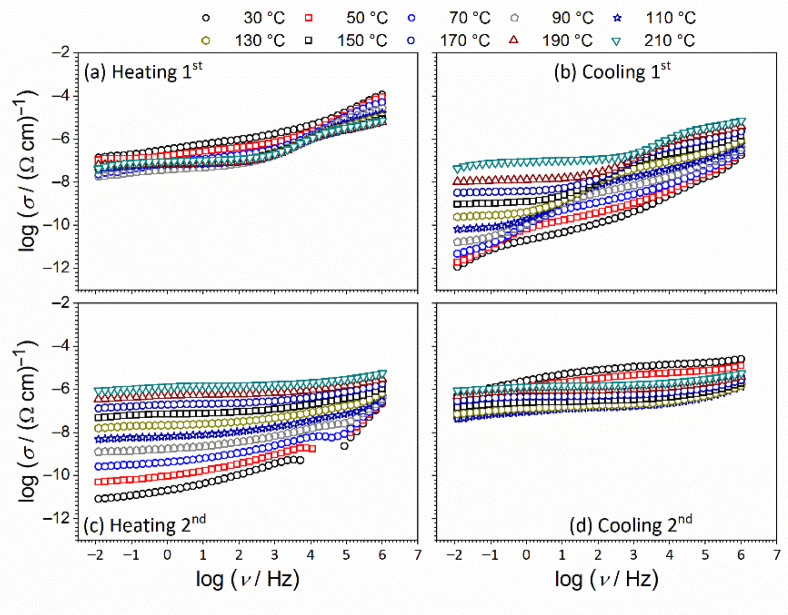
Conductivity spectra for CeO_2_ sample in form of a thick pellet (bulk) in: (**a**,**b**) 1st; and (**c**,**d**) 2nd heating/cooling runs, respectively.

Figure 6 shows conductivity spectra at different temperatures for the ceria sample in the form of a thick pellet in the heating/cooling cycles. The conductivity isotherms are similar in shape, and different spectral features can be observed. Firstly, it can be observed that heating runs (Figure 6a,c) show a restricted range and are random in terms of the temperature variation of conductivity in comparison to cooling runs (Figure 6b,d). In the latter, a nearly frequency-independent conductivity related to the long-range transport of charge carriers (DC conductivity) is observed at low frequencies, whereas with increasing frequency conductivity dispersion occurs in a power-law fashion due to their localized short-range motions. In the lowest-frequency region of spectra at highest temperatures, a decrease in conductivity is also visible due to the electrode polarization effect at the sample surface [34,35,36,37,38].

The temperature-dependent measurements were performed in a nitrogen atmosphere which resulted in different trends of DC conductivity in the heating/cooling cycles. In the heating runs (Figure 6a,c), the sample is dehydrated and water molecules become desorbed from the surface, while in the cooling runs (Figure 6b,d), the DC conductivity is thermally activated and increases with temperature showing semiconducting behavior, which was not the case in the heating runs [13,21,22].

**Figure 7:**
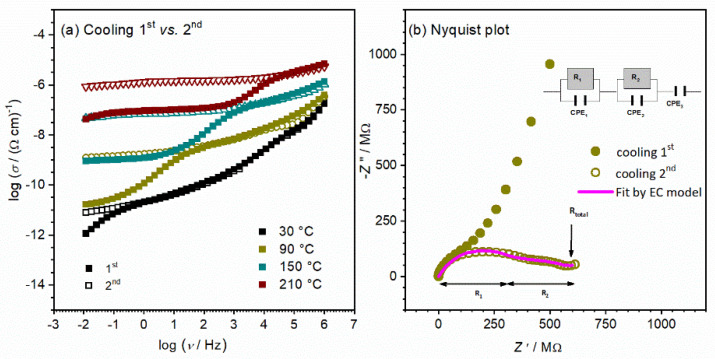
(**a**) Comparison of conductivity spectra at various temperatures in different cooling runs; and (**b**) complex impedance plane (Nyquist plot) for CeO_2_ bulk sample in the configuration of pellet at 90 °C.

The conductivity spectra for the CeO_2_ sample in the form of a thick pellet measured at 90 °C in the first and second cooling run are shown in Figure 7a. The effect of heating/cooling runs on structural relaxation and the effect on the electrical properties are observed. As mentioned earlier, the conductivity spectra mainly show two typical features: (I) a plateau at low-frequencies that corresponds to the DC conductivity, and (II) dispersion at higher frequencies which is related to the short-range transport of charge carriers. When cycling, the shape of conductivity dispersion changes. Indication of the additional process is observed for the first cooling run in the lower frequency range, which starts to be visible for 90 °C conductivity isotherms (Figure 7a). This additional process shifts along with temperature to higher frequencies. Moreover, in the second cooling run, the process at lower frequencies completely disappears from the spectra.

To shed more light on this interesting effect, the obtained experimental results are presented differently. The Nyquist plot for the CeO_2_ sample in the form of a pellet for the corresponding first and second cooling run at 90 °C is presented in Figure 7b. At first glance, it can be seen that the impedance spectra consist of two overlapping but still well-formed semicircles and a low-frequency spur. The presence of multiple semicircles/spurs can be related to different electrical processes in a material [39,40]. The spectrum of the second run shows lower values in comparison to the first, and different ratios of the observed processes. The initial step in the interpretation is to choose an appropriate electrical equivalent circuit (EEC) model and then estimate the parameters of the chosen model. The corresponding equivalent circuit model used for fitting the experimental data for the second run spectrum is shown in Figure 7b and fitting parameters are listed in Table 1.

According to the appropriate equivalent circuit model used, ongoing processes can be identified and separated based on the order of magnitude of the obtained fitting parameters [41,42,43]. The complex impedance spectrum is described by two parallel equivalent circuits (R-CPE) connected in series. An individual impedance semicircle can be represented by an equivalent circuit consisting of a resistor and capacitor connected in parallel. Ideally, such a semicircle passes through the origin of a complex plot and yields a low-frequency intercept on the real axis corresponding to the resistance, *R*, of the corresponding process. However, the experimental data obtained show a depressed semicircle whose center is below the real axis, and in such cases, a constant phase element (CPE) is used, instead of the ordinary capacitor in equivalent circuits. The CPE is an empirical impedance function of the type:

(1) $$Z^{*}{}_{CPE}=\frac{1}{A{\left(i\omega \right)}^{\alpha }}$$

where *A* and *α* are the constants. For the low-frequency spur, the third CPE element connected in series is added to the model. The parameters for each circuit element (*R*, *A*, and *α*) were obtained directly from the measured impedance data using the complex non-linear least square (CNLLSQ) fitting procedure.

The semicircle at higher frequencies corresponds to the sample bulk (equivalent circuit R1-CPE1), whereas the semicircle at low frequencies (R2-CPE2), is due to the grain boundaries effect. Additionally, the spur at low frequencies is connected with the surface-electrode effect. From the values of resistance, obtained from equivalent circuit modeling along with sample geometry, we evaluated the total DC conductivity (Table 2). From cycle comparison in Figure 7a,b, it can be clearly seen that processes that correspond to bulk have the same extent in both cycles. Furthermore, differences are obvious in the process related to the grain boundaries effect, which is present at lower frequencies as a result of the polycrystalline form of the CeO_2_ sample pressed in the form of a pellet. With cycling, this additional contribution to total DC conductivity is diminished by a few orders of magnitude but is still present in the second cooling run. It appears that better connectivity between the grains occurs with cycling, which has a positive impact on conductivity.

**Figure 8:**
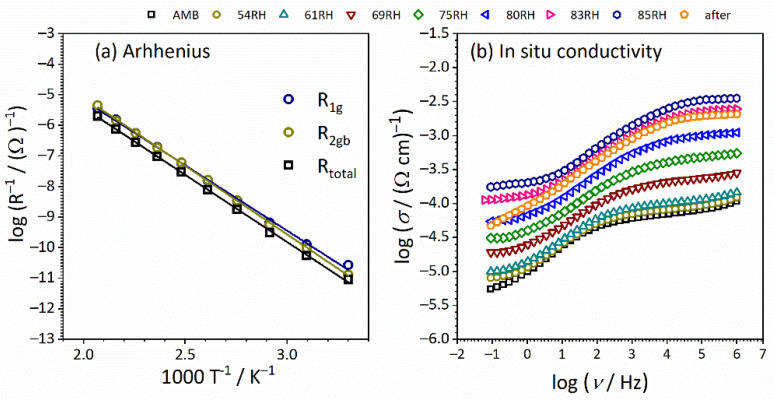
(**a**) Temperature dependence of grain, grain boundary, and total resistance (log(1/*R*) vs. 1000/*T*) for CeO_2_ sample in pellet form. Solid lines represent the least-square linear fits to experimental data; (**b**) Conductivity spectra at ambient temperature for CeO_2_ sample in form of pellet measured under controlled relative humidity (RH), from 30% (ambient) up to 85%.

Even though the above-proposed interpretation originally referred to ceramics, [42,43,44], it can be applied to any other solid materials with conductive crystalline grains and grain boundaries, which is further supported by the determined capacitance values presented in Table 1. The interpretation is directly connected to the volume fraction of each region present in the samples, i.e., small capacity values correspond to large volume fractions (bulk), while large capacity values are attributed to the phase with lower volume fraction. Therefore, order of magnitude values of fitting parameters obtained using equivalent circuit modeling allows the determination of the electrical properties of different regions within the electrode-material system. In general, low capacitance values correspond to the largest volume fraction, bulk, ~10^−12^ F, while higher values between 10^−11^ and 10^−8^ F are related to the processes with lower volume fractions. Hence, the obtained capacitance values in our study for sample CeO_2_ in pellet form of 3.70 × 10^−12^ F and 1.55 × 10^−10^ F are consistent with grain and grain boundary effects, respectively. Moreover, the activation energy for DC conductivity for the observed process (grain, grain boundary, and total) is calculated from the slope of log(1/*R*_x_) vs. 1000/*T* presented in Figure 8a and listed in Table 2. 

The values for the activation energy related to different processes (i.e., grain and grain boundaries) are close to each other, ranging from 0.87 to 0.90 eV. The approximated total DC conductivity at 90 °C is evaluated to be 2.5 × 10^−9^ (Ω cm)^−1^ using the equation: *σ*_DC_ = *d*/(*R*_total_ × *S*) and correlates to the range of semiconductors. Here, it is interesting to observe that the resistance of grain is at the same order of magnitude in comparison to the resistance related to the grain boundary (~10^8^), which results in a conductivity decrease of 0.3 orders of magnitude. Thus, we can see a slight blocking effect on long-range charge transfer which hampers the faster transport process in the crystalline grain interior.

#### 3.3.2. Thick Pellet Configuration: Humidity-Dependent Conductivity

In Figure 8b the conductivity spectra recorded in a wide relative humidity (RH) range, from 30% (ambient) to 85% for CeO_2_ sample in pellet form, is presented. The spectrum at ambient conditions (30% RH) is similar to the measurements for second heating run (see Figure 6c). One can see that high conductivity values are observed, which are related to the atmosphere and condition measurements. For impedance temperature measurements, nitrogen atmosphere has a negative effect on conductivity, which is the opposite if we are talking about ambient conditions and atmosphere (air). Under ambient conditions, water molecules are adsorbed on the surface and the sample is not dehydrated, resulting in the relatively high conductivity observed [45].

Under controlled humidity, with an increase in RH, the shape of the conductivity spectra remains the same. However, a shift to higher conductivity values along the y-axis is present. Humidity/moisture has a positive effect through a wide frequency range, at the same time on long as well as on short-range charge carriers transport. An interesting effect is observed after the measurements. The relaxation is slow and conductivity values need a long time to return to the initial values. This implies that thickness plays a crucial role in the relaxation process.

**Figure 9:**
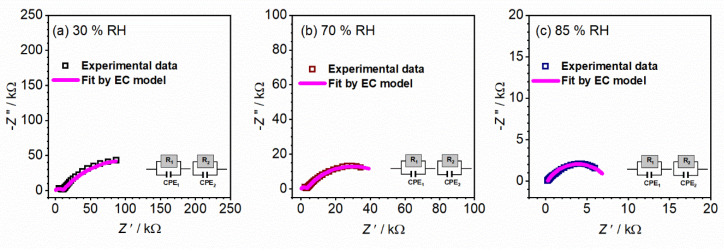
Complex impedance spectra for sample CeO_2_ sample in the form of pellet measured at various relative humidities (**a**) 30%, (**b**) 70%, and (**c**) 85%. The symbols (colored open squares) denote experimental values, whereas the solid magenta line corresponds to the best fit. The corresponding equivalent circuit model is composed of multiple parallel combinations of the resistor (R) and the constant-phase element (CPE), used for fitting the data of individual spectra. The goodness of the fit (i.e., chi-squared value) is in the range of 0.001–0.02.

To obtain more insight into present processes in controlled relative humidity environments, the impedance data are plotted as the Nyquist plot (see Figure 9). The corresponding equivalent circuit model used to fit the experimental data is shown in Figure 9 and fitting parameters are given in Table 3. The less pronounced semicircle at higher frequencies corresponds to the sample bulk (equivalent circuit R1-CPE1), whereas the dominant semicircle at low frequencies (R2-CPE2), is due to the grain boundaries/surface effects. The proposed interpretation is in line with calculated capacitance values of ~10^−11^ F and 10^−6^ F and related to bulk, along with grain and surface effects, respectively. From the values of determined resistance, along with sample geometry, the total DC conductivity is evaluated (see Table 3). One can see how the increase in humidity has a positive effect on the total DC conductivity, similarly to the temperature effect with semiconducting behavior. In the case of conductivity, an increase in RH from 30 to 85% results in a pronounced jump of one order and half in DC conductivity, from 3.81 × 10^−6^ (Ω cm)^−1^ to 9.91 × 10^−5^ (Ω cm)^−1^, respectively.

Furthermore, noted changes in the conductivity spectra could be described as a result of humidity changes, i.e., the moisture increase and impact on the processes observed and their ratios in the CeO_2_ sample in the form of a pellet. Thus, the concentration of charge carriers increases first on the surface of the sample, and with time the influence moves further through the sample, which can be seen by changes in the shape of spectra and corresponding calculated parameters (Figure 9 and Table 3). 

As the relative humidity increases, the high-frequency semicircle associated with the bulk process becomes less pronounced and shifts to the edge of the frequency range of the measurements. At the same time, the low-frequency semicircle starts to dominate the spectrum, which could clearly be seen at 85% RH. The change of humidity affects the process starting on the surface and moving inside the sample, and directly impacts the resistivity of the grain boundaries and capacitance values.

#### 3.3.3. Thin-film Configuration: Controlled Relative Humidity

**Figure 10:**
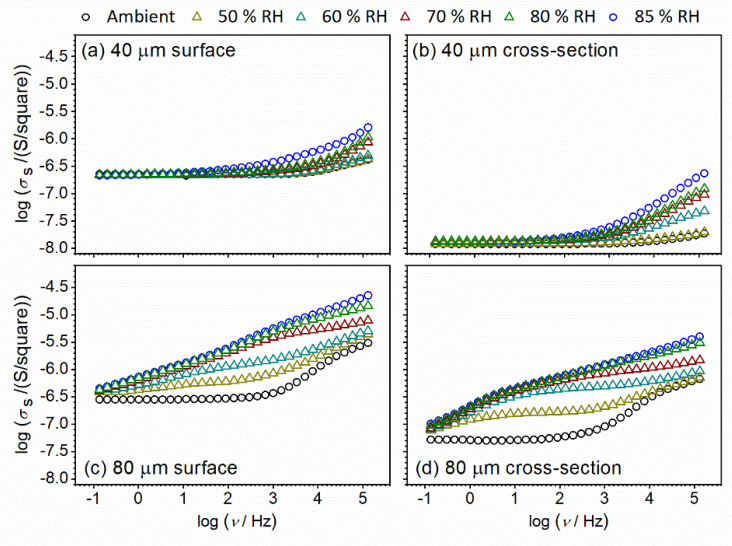
Conductivity spectra for CeO_2_ sample prepared in thin-film configuration with a thickness of: (**a**,**b**) 40 μm, and (**c**,**d**) 80 μm.

After a detailed investigation of the CeO_2_ sample in pellet form and its temperature-dependent electrical properties and sensitivity to the humidified environment, thin-film configuration and corresponding properties of the CeO_2_ sample were investigated. The state in mind should be changed from pellet (bulk) mode to thin-film (both surface and cross-section mode). As can be seen, the relaxation of the bulk sample is relatively slow, so the idea is to investigate the influence of sample thickness on sample properties. The conductivity spectra at ambient temperature for CeO_2_ thin-film samples with different thicknesses and setup (surface vs. cross-section) measured under controlled relative humidity (RH), from 30% (ambient) to 85%, are presented in Figure 10. The obtained results are interesting in a few aspects. For example, one can see that film thickness (i.e., 40 μm and 80 μm, respectively) has an impact on the shape of conductivity spectra. For different setups, the shape of the spectra also remains the same for each thickness under controlled relative humidity.

At ambient relative humidity (i.e., 30%), the DC surface conductivity is almost the same for 40 and 80 μm (Figure 10), having a value of ~2.25 × 10^−7^ (Ω square) which implies that film thickness does not directly influence surface conductivity of CeO_2_ samples under steady air atmosphere and ambient conditions, just the shape of spectra, as noted before. On the other hand, a comparison of spectra at ambient conditions for cross-section setup reveals an increase in DC conductivity by half an order of magnitude, when the thin-film thickness is increased from 40 to 80 μm. DC conductivity increases from 1.20 × 10^−8^ (Ω cm)^−1^ to 5.25 × 10^−8^ (Ω cm^)–1^. The observed effect of thickness is in line with further increase of conductivity at ambient conditions observed for the pellet of CeO_2_ sample having a value of total DC conductivity equal to 3.98 × 10^−6^ (Ω cm)^−1^ and a bulk DC conductivity of 6.92 × 10^−5^ (Ω cm)^−1^. A direct correlation between surface and cross-section values cannot be made due to the different geometry setups and consequent measuring units.

**Figure 11:**
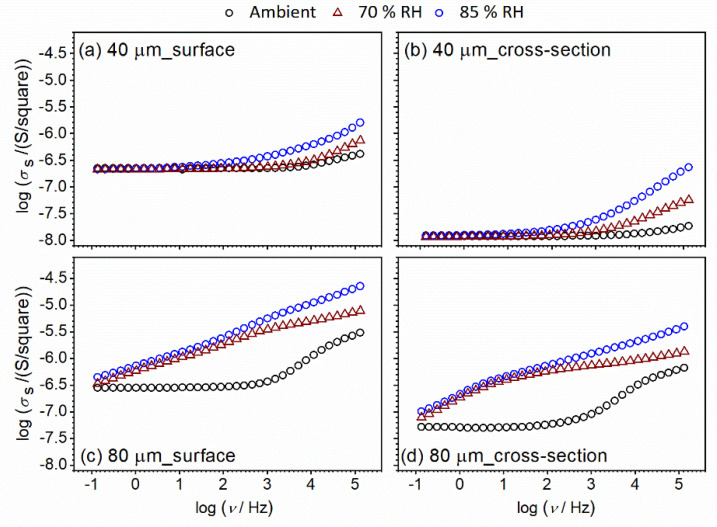
Conductivity spectra for CeO_2_ sample prepared in thin-film form with a thickness of: (**a**,**b**) 40 μm; and (**c**,**d**) 80 μm. Different configuration setup: (**a**,**c**) surface; and (**b**,**d**) cross-section, measured at ambient temperature and under controlled relative humidity (RH): ambient, 70% and 85%. Relative humidity stabilization is achieved with a saturated aqueous solution.

Interestingly, going back to the shape of the conductivity spectra, differences in spectra with changes in relative humidity for thinner thin-film samples are visible only at higher frequencies (dispersion part of spectra, which corresponds to localized short-range charge carriers) in both configurations (surface vs. cross-section) (Figure 10a,b). At the same time, the DC part of the spectra which corresponds to long-range charge carriers remains unaffected during exposure to a humidified environment. With an increase in thickness from 40 to 80 μm, conductivity starts to be sensitive to the RH conditions in both surface and cross-section configuration, through the wide measured frequency range (Figure 10c,d). Relative humidity influences, at the same time, long-range and localized short-range charge carriers. Moreover, in addition to in-situ measurements, we used another saturated aqueous solution (NaCl) and obtained saturated static values of 70% RH for the studied CeO_2_ samples, along with 85% RH. In this way, we can compare the conductivity spectra in in-situ and static modes. In Figure 11, conductivity spectra at two different static RH values, 70% and 85%, respectively, are presented. A good correlation is observed between 70% RH spectra obtained in in-situ and static mode.

**Figure 12:**
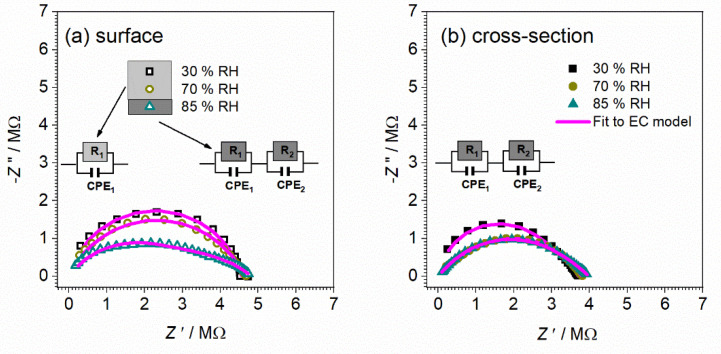
Complex impedance spectra for CeO_2_ sample prepared in thin-film form with a thickness of 40 μm measured at ambient temperature with different configuration setups: (**a**) surface and (**b**) cross-section, and under controlled relative humidity (RH): ambient—30%, 70%, and 85%. The symbols (colored open/full squares) denote experimental values, whereas the solid magenta line corresponds to the best fit. The corresponding equivalent circuit model is composed of multiple parallel combinations of the resistor (R) and the constant-phase element (CPE), used for fitting the data of individual spectra. The goodness of fit (i.e., chi-squared value) is in the range of 0.0002–0.006.

**Figure 13:**
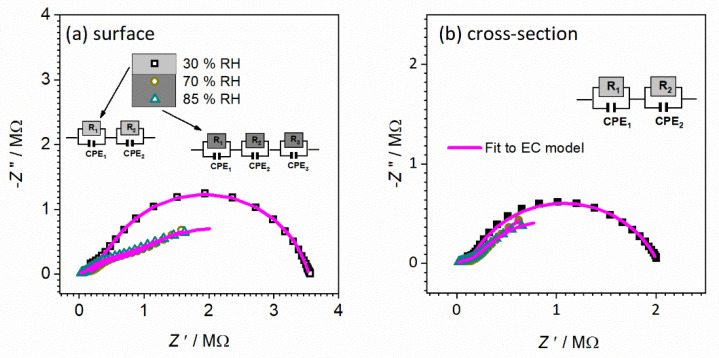
Complex impedance spectra for CeO_2_ sample prepared in thin-film form with a thickness of 80 μm measured at ambient temperature in: (**a**) surface, and; (**b**) cross-section configuration, under controlled relative humidity (RH): ambient—30%, 70%, and 85%. The symbols (colored open squares) denote experimental values, whereas the solid magenta line corresponds to the best fit. The corresponding equivalent circuit model is composed of multiple parallel combinations of the resistor (R) and the constant-phase element (CPE), used for fitting the data of individual spectra. The goodness of fit (i.e., chi-squared value) is in the range of 0.0003–0.001.

The observation that thicker film (80 vs. 40 μm) is, similarly to pellet, more sensitive to relative humidity, foreshadows once again a gradual transition from thin-film to bulk configuration with an increase in thickness. In an attempt to shed more light on the effect observed above on sensitivity to humidified environments, impedance data are presented in the Nyquist plot presentation (Figure 12 and Figure 13).

The corresponding equivalent circuit model used to fit the experimental data for the 40 μm CeO_2_ thin-film sample is shown in Figure 12 and fitting parameters obtained from equivalent circuit modeling of complex impedance spectra are given in Table 4.

For both surface and cross-section measurements, as can be seen, the total DC conductivity values (i.e., total resistance value) remain approximately the same, no matter what the RH conditions, which implies that the RH level does not affect the total resistance/conductivity. On the other hand, for surface and cross-section measurements, the shape of spectra and number of observed processes (semicircles) is gradually changing with higher RH conditions, i.e., a new process is present with moisture increase. For surface setup at ambient and 70% RH conditions, only one electrical process that can be confirmed is related to the process in bulk, which is in line with the calculated capacitance value ~10^−12^ F (Figure 12a). By increasing moisture up to 85% RH, a new process is observed with similar resistance and higher capacitance value which could be related to the effect of moisture on the surface of the thin-film sample. It is interesting to note at this point that the highest moisture level obtained did not affect the total conductivity but has a positive effect on the bulk process, which is visible in the decrease in the resistance value two-fold (from 4.6 to 2.4 × 10^6^ Ω) for a 40 μm thin-film sample measured in surface configuration.

Furthermore, the cross-section measurement configuration shows different trends. In contrast to the surface setup, for all RH conditions, two semicircles are observed related to charge transfer in bulk (high frequencies) and at grain boundaries (low-frequency) with calculated values of the capacitance of ~10^−12^ and ~10^−10^, respectively. The observed interpretation is in line with that for the pellet (bulk) CeO_2_ sample. Turning to the higher RH conditions (70 and 85%), the lower-frequency semicircle is slightly affected by the environment, as confirmed by the change in its shape and corresponding parameters. Due to an increase in RH level, the resistance and capacitance increased (see Figure 12b and Table 4). Based on the following processes and evaluation of complex impedance spectra, it can be concluded that surface measurements turn out to be more sensitive to relative humidity changes, emphasized for higher RH.

Moving forward to thicker thin-film samples, i.e., 80 μm CeO_2_, the corresponding equivalent circuit model used for fitting the experimental data is shown in Figure 13 and fitting parameters are given in Table 5. Again, for both surface and cross-section measurements, as can be seen, the total values of DC conductivity values (i.e., total resistance value) remain approximately the same, no matter which RH conditions, except for a small change in the cross-section measurement at the highest RH of 85%. Therefore, it implies that RH level does not affect the total resistance/conductivity, similarly to thinner, 40 μm thick thin-film. Moreover, for both setups at ambient RH, two semicircles are observed at high and low frequencies related to the processes in bulk and grain boundary, respectively.

This effect is similar to the measurements for sample in pellet form and 40 μm sample in cross-section setup where two processes are also observed: as in the 40 μm sample, in this configuration the positive effect of moisture is observed in the bulk process where the decrease in the resistance value is two-fold (from 4.6 to 2.4 × 10^6^ Ω) for a 40 μm thin-film sample measured in surface configuration. It seems that, with an increase in thickness, an additional process is also visible for surface set-up measurement. For the surface measurement setup (see Figure 13a), with an increase in RH from ambient level to 70% and 85%, the shape of spectra and number of observed processes (semicircles) gradually changes, i.e., a new process is present with an increase in moisture. This process can be seen in the Nyquist plot as an additional semicircle (the third) at the middle-low frequency range. Moisture for this setup has a strong effect and does not just influence grain boundary resistance and capacitance, but contributes to a new effect (process). An increase in RH level has a positive effect on the bulk process which is visible in the three-fold decrease in the resistance value (from 3.7 to 1.1 × 10^5^ Ω), similarly to the 40 μm thin-film sample measured in the surface configuration. On the other hand, the cross-section setup for 80 μm thick thin-film, see Figure 13b, as mentioned above, has two observed processes. Based on the presented results, we can conclude that surface measurement turns out to be more sensitive to relative humidity changes, emphasized for higher RH, along with an increase in thin-film thickness from 40 to 80 μm.

**Figure 14:**
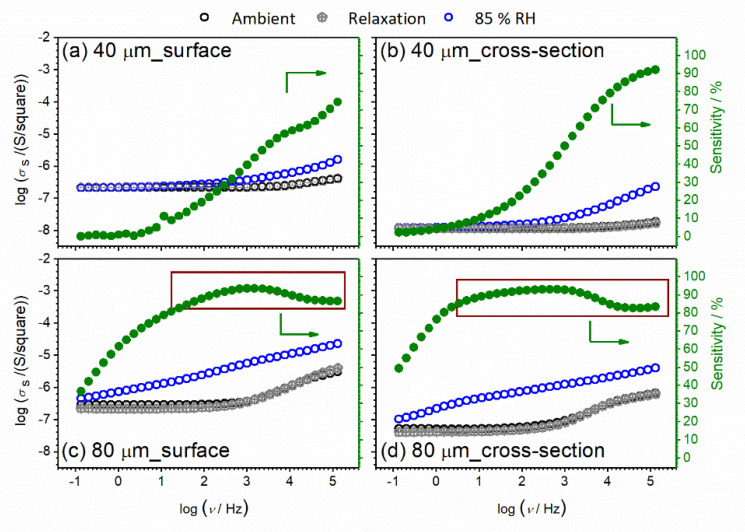
Conductivity spectra for CeO_2_ sample prepared in thin-film form with a thickness of (**a**,**b**) 40 μm; and (**c**,**d**) 80 μm. Different configuration setups: (**a**,**c**) surface; and (**b**,**d**) cross-section, measured at ambient temperature and under controlled relative humidity (RH): ambient—30%, 85% and relaxation to ambient after measurements.

We also study the relaxation process to ambient conditions for CeO_2_ thin-films samples after being exposed to high levels of RH. With moisture variation, the changes occur instantaneously (see Figure 14). The thickness of the film does not affect the relaxation process and remains instant. Therefore, moisture sensitivity is a reversible process for thin-film samples, in contrast to pellet form samples, and could furthermore be investigated with other thickness values to find the boundary for reversibility of humidity sensitivity. Once more, by the relaxation experiment, we showed that moisture directly affects conductivity spectra in the dispersion part, i.e., on the localized short-range charge carriers.

Taking the analysis further, we calculated the sensing response (SR) of the thin-film samples with different thicknesses. *SR* was calculated through the whole frequency range of impedance measurements using the expression *SR* = 100 × (*R*_0_ − *R*_85_)/*R*_0_, where *R*_0_ is the resistance of the sample at ambient conditions, before exposure to the water vapor (i.e., 30% RH), and *R*_85_ is the resistance at 85% RH. The humidity sensing response of the studied thin-film sensors is revealed in the right panel in Figure 14. As can be seen, different behavior is observed regarding the measurement configuration and thin-film thickness. In both configurations, humidity sensitivity of 80 um thin-film shows high values, *SR* > 80% above 1 Hz and 10 Hz for surface and cross-section, respectively. On the other hand, a constant increase is observed for the thinner sample through the whole frequency range. The observed behavior and trends indicate how the thickness and configuration setup along with the switch from bulk to thin-film play an important role in humidity sensing behavior.

## 4. Conclusions

Nano-powder of pure ceria was prepared. Pellets were made by pressing the prepared powders. Slurries were prepared and deposited at conductive glass substrates by tape casting method with nominal 10, 20, 40, and 80 μm thickness. Ceria powder yielded crystallites of about 23 nm while slightly greater spherical particles were observed on micrographs, with specific surface area of 38.7 m^2^g^−1^ along with a band-gap of 3.55 eV. Depth profiling of tape-casted ceria films shows homogeneous monophasic films with relative roughness of the order of 200 nm (for 80 μm film).

For a bulk sample, the impedance spectra consist of two overlapping, but still, well-shaped, semicircles and a low-frequency spur, corresponding to the sample bulk, the grain boundaries effect and the surface-electrode effect. It was found that, with cycling, there is better connectivity between the grains, which has a positive impact on conductivity.

Under controlled humidity, with an increase in RH, the shape of the conductivity spectrum stays the same, but a shift to higher conductivity values is present. Relaxation is slow and conductivity values need a long time to return to starting values suggesting that the thickness of the pellet plays a crucial role in the relaxation process. The increase in humidity has a positive effect on the total DC conductivity, similar to the temperature effect in semiconducting behavior.

Conductivity spectra at ambient temperature for CeO_2_ thin-film samples with different thicknesses and setup (surface vs. cross-section) were measured under controlled relative humidity (RH), from 30% (ambient) up to 85%. At ambient conditions, DC conductivity increases by half order of magnitude for the cross-section setup when the thin-film thickness is increased from 40 to 80 μm.

For the surface measurement setup (80 μm CeO_2_), with an increase of RH from ambient level to 70 and 85%, the shape of spectra and number of observed processes (semicircles) gradually changes, i.e., a new process is present along with moisture increase. We can conclude that surface measurement turns out to be more sensitive to relative humidity changes, emphasized for higher RH, along with an increase in thin-film thickness from 40 to 80 μm. Consecutively, moisture sensitivity is a reversible process for thin-film samples, in contrast to pellet form samples.

## Figures and Tables

**Table 1:** The fitting parameters were obtained from equivalent circuit modelling of complex impedance spectra measured at various temperatures for a CeO_2_ sample in the form of a pellet.

Parameters	Temperature /°C		
	90	150	210
*R*_1_/Ω	3.01 × 10^8^	5.09 × 10^6^	2.79 × 10^5^
*A*_1_/(s^α^ Ω^−1^)	4.05 × 10^−11^	6.06 × 10^−11^	7.97 × 10^−11^
*α*_1_	0.72	0.80	0.82
*C*_1_ */F	3.70 × 10^−12^	8.03 × 10^−12^	7.59 × 10^−12^
*R*_2_/Ω	3.15 × 10^8^	5.44 × 10^6^	2.20 × 10^5^
*A*_2_/(s^α^ Ω^−1^)	1.55 × 10^−9^	2.69 × 10^−9^	6.85 × 10^−9^
*α*_2_	0.47	0.59	0.62
*C*_2_ */F	1.55 × 10^−10^	1.43 × 10^−10^	1.28 × 10^−10^
*A*_3_/(s^α^ Ω^−1^)	1.53 × 10^−7^	6.28 × 10^−7^	5.14 × 10^−6^
*α*_3_	0.61	0.46	0.41

* Capacity (*C*) calculated from the equation: *C* = *A*(*ω*_max_)*^α^*^−1.^

**Table 2:** DC conductivity, *σ*_DC_, the activation energy of total DC conductivity, *E*_DC_, and activation energies for each particular contribution (grain and grain boundary) for the CeO_2_ sample in the form of a pellet.

CeO_2_ Sample—Pellet Form	
*σ*_DC_^a^/(Ω cm)^−1^	1.22 × 10^−9^
*E*_DC_/eV	0.88
*E*_g_/eV	0.87
*E*_gb_/eV	0.90

^a^ DC conductivity at 90 °C.

**Table 3:** The fitting parameters obtained from equivalent circuit modeling of complex impedance spectra measured at various relative humidities (RH) for CeO_2_ sample in pellet form.

Parameters	Relative Humidity (RH)		
	30%	70%	85%
*R*_1_/Ω	10901	3345	292
*A*_1_/(s^α^ Ω^−1^)	1.43 × 10^−8^	2.49 × 10^−8^	4.61 × 10^−8^
*α*_1_	0.56	0.61	0.63
*C*_1_ */F	1.46 × 10^−11^	6.14 × 10^−11^	6.35 × 10^−11^
*R*_2_/Ω	1.87 × 10^5^	5.25 × 10^4^	7.31 × 10^3^
*A*_2_/(s^α^ Ω^−1^)	5.07 × 10^−6^	7.71 × 10^−6^	2.05 × 10^−5^
*α*_2_	0.55	0.58	0.64
*C*_2_ */F	4.85 × 10^−6^	4.01 × 10^−6^	7.05 × 10^−6^
*σ*_DC_^a^/(Ω cm)^−1^	3.81 × 10^−6^	1.35 × 10^−5^	9.91 × 10^−5^

* Capacity (*C*) calculated from the equation: *C* = *A(ω_max_)^α^*^−1. a^ DC total calculated from the sum of *R*_1_ and *R*_2_.

**Table 4:** The fitting parameters obtained from equivalent circuit modeling of complex impedance spectra measured at various relative humidity (RH) for CeO_2_ sample in the form of thin-film 40 μm: surface and cross-section.

Parameters	Surface Setup/RH%			Cross-Section/RH%		
	30	70	85	30	70	85
*R*_1_/Ω	4.57 × 10^6^	4.64 × 10^6^	2.37 × 10^6^	2.71 × 10^6^	1.84 × 10^6^	2.49 × 10^6^
*A*_1_/(s^α^ Ω^−1^)	1.41 × 10^−11^	7.83 × 10^−11^	6.23 × 10^−10^	4.30 × 10^−12^	7.48 × 10^−10^	1.81 × 10^−9^
*α*_1_	0.83	0.72	0.63	0.94	0.72	0.66
*C*_1_ */F	1.95 × 10^−12^	3.60 × 10^−12^	1.36 × 10^−11^	2.08 × 10^−12^	5.77 × 10^−11^	1.12 × 10^−10^
*R*_2_/Ω	/	/	2.45 × 10^6^	1.00 × 10^6^	1.98 × 10^6^	1.51 × 10^6^
*A*_2_/(s^α^ Ω^−1^)	/	/	2.12 × 10^−8^	1.44 × 10^−9^	3.06 × 10^−9^	2.76 × 10^−8^
*α*_2_	/	/	0.45	0.65	0.52	0.45
*C*_2_ */F	/	/	5.71 × 10^−10^	4.25 × 10^−11^	2.75 × 10^−11^	5.68 × 10^−10^

* Capacity (*C*) calculated from the equation: *C* = *A(ω_max_)^α^*^−1.^

**Table 5:** The fitting parameters obtained from equivalent circuit modeling of complex impedance spectra measured at various relative humidities (RH) for CeO_2_ sample in the form of thin-film 80 μm: surface and cross-section.

Parameters	Surface Setup/RH%			Cross-Section/RH%		
	30	70	85	30	70	85
*R*_1_/Ω	3.67 × 10^5^	1.59 × 10^5^	1.05 × 10^5^	1.25 × 10^5^	1.58 × 10^5^	2.87 × 10^5^
*A*_1_/(s^α^ Ω^−1^)	2.15 × 10^−10^	2.25 × 10^−10^	5.54 × 10^−8^	6.48 × 10^−12^	5.93 × 10^−8^	7.43 × 10^−7^
*α*_1_	0.71	0.76	0.44	0.98	0.368	0.29
*C*_1_ */F	4.54 × 10^−12^	8.87 × 10^−12^	7.92 × 10^−11^	4.87 × 10^−12^	1.95 × 10^−11^	1.69 × 10^−8^
*R*_2_/Ω	3.17 × 10^6^	6.72 × 10^5^	6.00 × 10^5^	1.89 × 10^6^	1.93 × 10^6^	1.32 × 10^6^
*A_2_*/(s^α^ Ω^−1^)	2.13 × 10^−10^	8.14 × 10^−8^	8.89 × 10^−8^	1.82 × 10^−9^	1.08 × 10^−6^	1.13 × 10^−6^
*α*_2_	0.83	0.54	0.58	0.72	0.70	0.69
*C*_2_ */F	4.78 × 10^−11^	6.85 × 10^−9^	1.06 × 10^−8^	2.00 × 10^−10^	1.43 × 10^−6^	1.35 × 10^−6^
*R*_3_/Ω	–	3.11 × 10^6^	2.98 × 10^6^	–	–	–
*A*_3_/(s^α^ Ω^−1^)	–	4.47 × 10^−7^	4.42 × 10^−7^	–	–	–
*α*_3_	–	0.59	0.55	–	–	–
*C*_3_ */F	–	5.62 × 10^−7^	5.54 × 10^−7^	–	–	–

* Capacity (*C*) calculated from the equation: *C* = *A*(*ω*_max_)*^α^*^−1^.

## Data Availability

Data Availability Statement: Data can be available upon request from the authors.
